# Decision support tool and suggestions for the development of guidelines for the helicopter transport of patients with COVID-19

**DOI:** 10.1186/s13049-020-00736-7

**Published:** 2020-05-25

**Authors:** Per P. Bredmose, Monica Diczbalis, Emma Butterfield, Karel Habig, Andrew Pearce, Svein Are Osbakk, Ville Voipio, Marcus Rudolph, Alistair Maddock, John O’Neill

**Affiliations:** 1 LifeFlight Retrieval Medicine, PO box 15166, City East, QLD 4002 Australia; 2 Air Ambulance Department, Oslo University Hospital, Oslo, Norway; 3 Norwegian Air Ambulance Foundation, Oslo, Norway; 4 NSW Ambulance Aeromedical Sydney HEMS, Sydney, Australia; 5 MedSTAR Emergency Medical Retrieval, Adelaide, Australia; 6 Centre for pre-hospital emergency care, Oulu University Hospital, Oulu, Finland; 7 DRF Stiftung Luftrettung gAG, Filderstadt, Germany; 8 Emergency Medical Retrieval Service, ScotSTAR, Paisley, Scotland; 9 Department of Emergency Medicine, Cairns Hospital, 165 The Esplanade, Cairns, QLD Australia

**Keywords:** COVID-19, Transport, Retrieval, Infectious disease, HEMS, Helicopter emergency medical service, Aeromedical transport

## Abstract

The novel coronavirus SARS-CoV2 emerged in December 2019 and is now pandemic. Initial analysis suggests that 5% of infected patients will require critical care, and that respiratory failure requiring intubation is associated with high mortality.

Sick patients are geographically dispersed: most patients will remain in situ until they are in need of critical care. Additionally, there are likely to be patients who require retrieval for other reasons but who are co-incidentally infected with SARS-CoV-2 or shedding virus.

The COVID-19 pandemic therefore poses a challenge to critical care retrieval systems, which often depend on small teams of specialists who live and work together closely. The infection or quarantining of a small absolute number of these staff could catastrophically compromise service delivery.

Avoiding occupational exposure to COVID-19, and thereby ensuring service continuity, is the primary objective of aeromedical retrieval services during the pandemic. In this discussion paper we collaborated with helicopter emergency medical services(HEMS) worldwide to identify risks in retrieving COVID-19 patients, and develop strategies to mitigate these.

Simulation involving the whole aeromedical retrieval team ensures that safety concerns can be addressed during the development of a standard operating procedure. Some services tested personal protective equipment and protocols in the aeromedical environment with simulation. We also incorporated experiences, standard operating procedures and approaches across several HEMS services internationally.

As a result of this collaboration, we outline an approach to the safe aeromedical retrieval of a COVID-19 patient, and describe how this framework can be used to develop a local standard operating procedure.

## Introduction

The SARS-CoV-2 virus, pandemic since March 2020 [1], causes the clinical syndrome of Coronavirus Disease 2019 (COVID-19) [2–4]. Most infections are mild or asymptomatic and upper respiratory tract symptoms, when present, are responsible for droplet spread of the pathogen. However, in some patients, especially the elderly, or those with cardiovascular or respiratory comorbidities [3, 4], infection causes respiratory failure, with potentially severe hypoxia.

Aeromedical retrieval services will be involved in treating and transporting patients with known or suspected COVID-19. This will primarily be those who are severely affected and require intensive care support [5], and patients with other pathology who are incidentally co-infected.

This article outlines the planning that such organizations should undertake prior to retrieving patients who may have COVID-19, and suggests a model for the management of severe cases of COVID-19 on helicopters.

Our understanding of COVID-19 is evolving daily, limiting our capacity to provide definitive evidence-based advice. Therefore, our consensus recommendations are based on our present knowledge of COVID-19; existing standard operating procedures for aeromedical transport; and lessons learned from managing similar pathogens (e.g. SARS, MERS).

Consensus was sought across an international range of high-volume retrieval services, by sharing of clinical practice standards, standard operating procedures (SOPs), and checklists. Our subsequent collaboration focussed on identifying areas of agreement and highlighting points of consensus or controversy (a description of the authors’ services can be found in Additional file 1).

The COVID-19 planning rubric described in this article can potentially be applied to any critical care retrieval service. The majority of COVID-19 patients will be transported by ground ambulances and paramedics, and although many of the same concerns may apply, the scope of this paper is limited to transport on rotary wing platforms.

Our objectives were to:

- Describe an approach to the selection of patients for transport, and the design of effective infection control plans.
- Outline key decisions and preparation that must be undertaken prior to transporting COVID-19 patients, particularly resolving conflicts between aeromedical SOPs and infection control strategies at various stages of the retrieval process.
- Combine available evidence and the expertise of the aviation, paramedical and medical teams to resolve such conflicts, and thereby mitigate risk.

At all stages, our decision making was intended to minimise risk to the team; risk to the patient; and risk to service continuity; in descending order of priority.

## Methods

In February and March 2020, systematic literature searches were performed on Pubmed, Embase, Ovid and the Cochrane Database to identify studies pertaining to aeromedical transfer of patients with COVID 19 or highly infectious diseases. Our search strategy is outlined in Additional file 2.

One relevant review article was found [6], summarising the literature pertaining to the aeromedical transport of highly contagious patients, predominantly by teams using specialist containment equipment. We drew upon this article to generate subject headings, subsequently adding issues we identified through our simulations and collaboration.

We invited input from a number of international HEMS services with whom we had an extant relationship, and used an informal collaborative process whereby all authors contributed and reviewed one another’s input. Amongst respondents, some HEMS services were in the process of developing an SOP. Other services, with access to alternative transport platforms, were planning to not transport COVID-19 cases by air to reduce risks to staff.

Several services have utilised a full crew walk-through simulation of a potential COVID-19 patient transport to identify weaknesses in existing plans and to optimize efficiency and safety [6]. Although there are no accepted reference standards or development templates for a decision support tool, we have used the AGREE standard for guideline development where possible [7].

## Results

### Selection of patients for transport

The core principle is to follow usual aviation and medical pathways where possible, with the additional goal of limiting staff exposure to COVID-19.

There should be a risk assessment at a regional/institutional level, preferably with retrieval clinician and crew input, which culminates in clear agreement on the circumstances in which it is appropriate for a service to undertake transfers of confirmed COVID-19 cases, and what precautions should be taken in patients who do not have confirmed infection. This process will evolve alongside the pandemic.

Key considerations in this risk assessment include:

- Vehicle availability. Preparing and decontaminating vehicles compromises vehicle availability, and alternatives to aeromedical transport (or transporting the retrieval team to the patient by helicopter and returning by ambulance) should be considered.
- Increased risk of occupational transmission in an enclosed aircraft cabin. Aerosolizing procedures like Non-Invasive Ventilation (NIV), high-flow nasal oxygen therapy and nebulization may pose high risks particularly in confined cabins [8]. Particular concern has been raised that NIV might raise the risk of nosocomial infection with SARS-CoV2 [9]. Patients requiring these therapies may require alternative management during transport, and a low threshold for intubation is advocated [8].
- Management of asymptomatic patients. Personal correspondence with other retrieval services has suggested that HEMS crew members have inadvertently been exposed to SARS-CoV-2 by patients in whom the initial tasking seemed unrelated to respiratory disease. Some retrieval services have therefore suggested that all patients transported during this pandemic should wear a surgical mask if spontaneously breathing, due to their proximity with the crew and the potential for a mask to limit droplet spread [10]. In areas with community transmission, if patients require NIV or High-Flow Nasal Oxygen, transport by road rather than helicopter should be considered, even if the patient is deemed low risk for COVID-19. Turnaround times for testing for COVID-19 may be delayed in rural areas: in this context all patients with respiratory compromise should be treated as potential COVID-19 patients. Aeromedical services may have a role in expediting testing for COVID-19 by delivering and collecting sample kits from rural areas.
- Service continuity. The pandemic will inevitably result in staff infections or isolations. Almost 9% of COVID-19 cases in Italy have been in healthcare workers [11]. This may disproportionately impact HEMS services, which are reliant on a small group of highly trained individuals who are not easily replaceable.

### Infection control strategies

#### Medical personal protective equipment (PPE)

An adequate supply of PPE must be assured: most HEMS bases would not normally stock the quantities needed to allow the transport of multiple patients with strict droplet or airborne precautions. Local shortages of PPE are likely to be a significant challenge to maintaining service delivery.

All staff must be trained in the appropriate use of PPE, including donning and doffing procedures [12]. Staff are likely to need practice in applying, wearing, working in, and disposing of PPE. For example, cockpit communications systems may be less effective with a mask in situ: low fidelity task simulation as well as full scale simulation are useful tools for practice [13].

PPE guidelines vary between jurisdictions, and these must be applied with heed paid to aviation regulations, practicality, and risk to staff.

Generally speaking however, PPE guidelines make a distinction between droplet-level and aerosol- (or airborne-) level precautions. The former usually includes a surgical facemask, impermeable gown, eye protection, and double gloves. The latter upgrades respiratory protection to a higher level of filtration, either N95/FFP2 (such as in Australia) or N99/FFP3 (UK/Germany) facemask, and may also include a full-body suit rather than a gown terminating at the shins. Shoe/boot covers, or washable wellington-style boots, can be helpful additions. A full fluid-repellent suit may be more practical than a surgical gown for windy conditions outside.

The risk of aerosols once a patient is intubated and adequately anaesthetised is low [14], likely occurring only if there is an inadvertent extubation, circuit disconnection proximal to an HME filter, or a need for open endotracheal suctioning. The former two events are rare, the latter can be mitigated by the use of closed suction systems, but a single such event may expose staff to an aerosol with a high viral load and potentially higher risks of infection and severe disease.

Aerosol-level PPE, as described above, may be incompatible with usual rotary flight PPE, particularly flame-retardant flight suits, helmets, and lifejackets. Services must therefore make pragmatic decisions about the feasibility of different levels of PPE in different transport environments. Options include:

- Using alternative transport platforms, eg land or fixed-wing.
- Precluding certain procedures in flight.
- Using droplet-level PPE for flight if a patient is felt to have a low risk of aerosol generation in transit.
- Wearing aerosol-level PPE at the expense of usual aviation PPE.
- Using portable commercial available isolation units such as the IsoArk or EpiShuttle devices. These allow patients to be sealed in a negative-pressure environment for transfer and will contain any aerosols. However, most aeromedical retrieval services will not have these available or be trained in their use.

The authors do not feel able to make a specific recommendation on the level(s) of PPE appropriate for the various phases of patient transfer, as their own services have made different decisions in context.

There is no firm consensus on whether PPE is needed for cockpit crew if the patient is intubated and it is possible to separate the cabin from the cockpit: some services advise that it is unnecessary, whereas others still advocate the use of PPE by the pilot and other cockpit crew [15].

#### Reducing exposure: people

The Centers for Disease Control and Prevention pragmatically suggest limiting the number of personnel who are in contact with a COVID-19 patient [16]. It may be possible to omit one or more members of a crew: for example, in daylight, with good weather, it may be appropriate to operate as a single- rather than dual-pilot aircraft. Such variations will occur at pilots’ discretion.

Ideally flight crew should not have any patient contact – this may contradict usual loading/unloading procedures, and so may require additional help from the referring and receiving sites. This should be discussed during the planning phase.

It may be possible to erect a physical divider between the cockpit and cabin, depending on flight regulations and aircraft configuration.

#### Reducing exposure: equipment

Many aeromedical retrieval aircraft routinely carry search and rescue equipment. Removing this, or placing it in protective bags or sheeting, limits contamination and simplifies decontamination. However, this may affect the aircraft’s operational abilities.

Similarly, it may be possible to minimise the amount of contamination by removing medical equipment not needed for the mission in question.

### Key decisions: at the referring hospital

The route taken through the hospital should be chosen to minimize the risk of cross-contamination. Prior coordination regarding arrival time can help to ensure smooth transit, with “clean” hospital staff opening doors. Identify the time and place to receive handover and don PPE (if the patient is in an isolation room, this should be before entering the room). Hospital-provided PPE may be preferred for initial packaging to preserve air ambulance PPE for transport.

#### Intubation

Intubation is an aerosolizing procedure, and so should be undertaken with additional precautions. Multiple best practice guidelines have been published [8, 14, 17–19]. Planning should focus on reducing potential aerosolization, minimizing the number of staff involved, and limiting the amount of equipment which will be contaminated, as well as ensuring the correct PPE for each staff member. We have attached a guide to intubation, written specifically for retrievalists, drawing upon the guidelines referenced above (Additional file 3).

#### Packaging

Consider wiping down the patient after intubation to remove droplets from their skin and reduce their infectivity. Package the patient in a clean sheet, surgical drape or plastic wrap. Transporting prone patients is feasible; however, we suggest that services only undertake prone transfers if they are familiar with the process.

#### Ground handling

The number of staff handling the patient should be minimized and ideally limited to only those involved in patient care. If weather permits, return to the aircraft via an open-air route, rather than hospital corridors. It may be prudent for hospital staff who have already been in contact with the patient to assist with loading and unloading rather than exposing others.

Similarly, when offloading, direct admission to ICU is preferable to going via other areas. If intensive care staff come to the helipad, they can accompany the team through the hospital. Remaining retrieval staff may be able to decontaminate the aircraft and doff their PPE. If this is a change to normal practice, agreements need to be made before the first transfer takes place. Prior to circuit disconnection, clamp the endotracheal tube in exhalation to avoid inadvertent aerosolization. We suggest attaching a clamp to the ventilator to ensure it is always immediately available.

#### Key decisions: in flight

Non-intubated patients should be asked or assisted to clean their hands before they enter the cabin to minimize surface contamination.

Ensure intubated patients have deep sedation and muscle relaxation to prevent coughing. Re-check the cuff pressure is adequate at altitude to avoid leaks. Take care to tighten (and consider taping) all airway connections to prevent inadvertent disconnections in transit.

Airway connections can be loosely wrapped in absorbent material, e.g. incontinence sheets, to minimise the impact of inadvertent disconnection. Consider tying and taping the endotracheal tube to the face to aid airway security.

### Key decisions: at mission completion

#### Waste disposal planning

There is likely to be an increase in clinical waste generated [15]. Clinical waste bins in hospitals contain up to 60% non-clinical waste [20]. Ensuring appropriate sorting of uncontaminated waste (e.g. clean packaging) and contaminated waste (e.g. used PPE), at the point of disposal can significantly reduce the amount of clinical waste generated.

Doffing PPE incurs a high risk of self-contamination [21]. A “spotter buddy” when doffing PPE will assist clinicians to doff PPE safely and correctly dispose of waste. Clinicians may be inclined to obviate the risk of contaminating domestic waste by denoting all waste as clinical waste. Patient equipment should be cleaned prior to exiting the receiving unit.

#### Decontamination

Cleaning and disinfection of surfaces should take place immediately following patient transport, and ideally be performed only by staff who have had close contact with the patient. Staff travelling in the cabin may not doff PPE until the cabin has been decontaminated, and those cleaning the aircraft should wear PPE for contact and droplet precautions (including a long-sleeved impervious gown, surgical mask, goggles and non-sterile gloves). Therefore, it may be prudent for medical staff to clean the cabin at the receiving hospital, prior to doffing their PPE (especially if there are PPE shortages). The cleaning procedure is specific to each aircraft but includes wiping of all surfaces with a cleaning product, then disinfectant or a combined product. If the aircraft is cleaned at the hangar, a PPE disposal system must be present.

Hospital-grade disinfectants used for norovirus will eliminate SARS-CoV-2, but it is essential to consult aircraft engineers, as some cleaning products may damage the aircraft. There therefore may be substantial differences in the way aircraft decontamination takes place among different services [22]. The aircraft will need additional time offline in order to be adequately cleaned and dried before a subsequent mission [23]. Wiping down the cabin is estimated to take an hour; additional time may be needed for fumigation and drying. A checklist of areas to be cleaned can be used to improve coverage.

### Key decisions: in the community

#### Accidental exposure

HEMS crew members should practice social isolation and meticulous hand hygiene out of work hours to limit community exposure to COVID-19. Crew members should self-isolate and test according to local guidelines in the event of known or suspected exposure. An accurate log of people who have had contact with COVID-19 patients should be kept by the retrieval service to allow contact tracing, and explicit information given to staff about sentinel symptoms.

#### Base living

For retrieval services where multiple teams work, eat and sleep on base, it is vital to manage the risk of cross-infection. Dalton et al. recommends introducing pre-emptive low cost social distancing measures in order to reduce the risk of spreading COVID-19 in households and workplaces [24].

Practical steps include

- Reducing/eliminating base visitors
- Limiting building access/egress to funnel staff though hand hygiene stations
- “Welcome if you’re well” signage, a no handshake policy. Gamifying no face touching [24]
- Segregating areas for multiple teams to work and rest, to limit cross contamination. Separate tables for eating
- Impervious mattress protectors on bedding. Staff to supply pillows and bedding rather than sharing
- Enhanced cleaning of surfaces in shared areas like bathrooms, kitchens and bedrooms.
- Post shift cleaning of all equipment and shared surfaces like work desks, computers, service phones etc.
- Limiting non-essential training; and shifting all meetings to video or teleconference
- Avoiding sharing food

### Decision support tool

Responding to COVID-19 requires services to: identify threats; generate potential solutions to them; choose which solutions are best; and implement them. This cycle will be repeated as the pandemic evolves.

We have developed a list of pertinent issues, which we present below as a decision support framework, designed to prompt local discussion and solutions (Table 1). Retrieval services are heterogeneous, and so use of the framework may generate different answers in different circumstances.

**Table 1:** Decision support framework

Theme	Topic	Considerations
Selection of patients for transport	Regional risk assessment	Vehicle availability. Availability of alternative platforms.
		Risk of occupational infection on different platforms.
		HEMS continuity and the impact of suspending HEMS/aeromedical services and redeploying staff.
		Planned management of patients without COVID-19 symptoms
Infection control strategies	PPE	PPE supplies
		PPE training (including aircrew)
		PPE compatibility with aviation safety and communications equipment
		PPE compatibility with working environment (eg heat stress, visibility)
	Minimise exposure: people	Protecting the flight crew from patient contact (screens, no patient handling)
		Reducing flight crew numbers
		Aligning medical/aviation shift patterns to avoid exposure of multiple crews
	Minimise exposure: equipment	Removal of extra medical/rescue equipment
		Removal of some aviation safety gear
		Packaging equipment in wipe-down packaging
		Choosing a route through the hospital to minimise cross-contamination
At the hospital	Accessing the patient	Time and place for handover and donning PPE
		Need for extra staff on ground (e.g. a runner in clean PPE to open doors/operate lifts)
	Intubation	Logistics of performing intubation in PPE
		Policy for use of PPE during the intubation of non-COVID-19 patients during pandemic COVID-19
	Packaging	How to package the patient to reduce their infectivity
At mission completion	Waste disposal	Management of increased clinical waste
		Sorting of waste to minimise clinical waste
	Decontamination	Suitable aircraft cleaning products, and their availability
		Where and by whom the aircraft will be cleaned
	Follow-up	Management of PPE breaches
		Management of exposed or symptomatic staff
In the community	Base living	Modifications to cleaning schedule
		Minimising staff on base
		Maintaining morale
		Social distancing off-duty

Table 1. Helicopter Emergency Service (HEMS), Personal protection equipment (PPE).

## Discussion

Decisions about whether or not to retrieve a patient during a pandemic must balance the risks and benefits to the patient, referring and receiving hospitals and retrieval teams. It is important for systems to plan how their resources can best be used in the pandemic context.

Regional guidelines and discussions involving referring and receiving clinicians, and retrieval teams, will help ensure clarity about whom to move, and when to move them, particularly as resources become stretched.

We believe that patients in whom inter-hospital aeromedical transport is being considered for COVID-19 should have up-to-date advance care directives, with conversations about them enacted early in a clinical episode. Tele- and videoconferencing systems may facilitate discussion between patients, referring, retrieving, and receiving clinicians.

HEMS systems, and pre-hospital systems, are heterogenous. Historically, the transport of patients with highly contagious diseases has usually been conducted by specialist, teams, utilising isolation pods and other specialised equipment [25]. That finite resource would be overwhelmed by the pandemic: all services must now prepare to safely transport COVID-19 patients, using available resources.

This paper provides a decision-making framework which services can apply to ensure that their plans address the main challenges in transporting COVID-19 patients. SOPs and guidelines for the transport of COVID-19 patients must be re-evaluated as knowledge of the disease evolves. This is a demanding task, and an additional burden to staff already fatigued by clinical work. We believe that social media, open access journals, and other free-to-access platforms are important for sharing clinicians’ experiences; collaborations on such platforms will facilitate the evolution and spread of context-specific best practice. Each service can access this shared pool of information and apply it to its specific context [26].

The management of COVID-19 patients involves an entire health system, much of which is not normally familiar with the logistics and mechanics of patient transfers. However, it is of utmost importance that those involved accept and understand the steps required to safely transport COVID-19 patients. Frequent liaison between prehospital and hospital services is important. In some circumstances, roles and responsibilities may change from daily practice, with prehospital and hospital staff assisting one another to ensure appropriate management of their patients.

PPE is only reliable if used correctly. Frequent organised teaching and practice in the use of PPE should be offered to all members of staff that are expected to use PPE.

The primary aim of a critical care retrieval service is to bring critical care knowledge, skills and equipment to patients who are disadvantaged geographically. In altering our regular practice during a pandemic, we must be careful to avoid compromising our usual standard of care. Simulation is a useful tool for identifying weaknesses in the flow of a patient through a system. We strongly advocate the use of simulation to help identify such oversights, as well as in preparing staff and systems for treating such patients [6].

## Conclusion

The transport of patients with known or suspected COVID-19 poses many challenges. All critical care retrieval services should anticipate the need to transfer patients during the pandemic, and formulate a local response to these challenges as soon as possible. We believe that the correct use of appropriate PPE, robust procedures for safe anaesthesia, and the use of simulation all contribute to enhanced crew and patient safety. Further evaluation and research should be conducted as the pandemic evolves so that we can learn more about how to safely move critically unwell patients by air.

## Supplementary information

**Additional file 1.**

**Additional file 2.**

**Additional file 3.**

## Data Availability

The search strategies and results of this is available as Additional file 2.

## Abbreviations

HEMS: Helicopter emergency medical service
PPE: Personal protective equipment
SOP: Standard operating procedure
NIV: Non-invasive ventilation
HEPA: High-efficiency particulate air (filter)
BVM: Bag-valve-mask
PEEP: Positive end-expiratory pressure
ETT: Endotracheal tube
RSI: Rapid sequence induction/intubation
