# Supplementary material for: Decision support tool and suggestions for the development of guidelines for the helicopter transport of patients with COVID-19
Source: Scand J Trauma Resusc Emerg Med. 2020 May 25;28:43. doi: 10.1186/s13049-020-00736-7 (PMC7247287; doi:10.1186/s13049-020-00736-7)
Supplement: Supplementary file 1 — Additional file 1. [file 13049_2020_736_MOESM1_ESM.docx]

# Appendix A

NSW Ambulance Aeromedical Sydney HEMS
NSW Ambulance Aeromedical Sydney HEMS is a critical care retrieval service based in New South Wales, Australia. Operating out of four bases, with helicopter, fixed wing and road retrieval platforms and performing around 3,000 critical care transports and prehospital missions per year with critical care paramedic/nurse and physician-based teams including winch-access prehospital trauma through to ECMO and IABP transfers.

MedSTAR Adelaide
MedSTAR is the single statewide emergency medical retrieval service for South Australia providing neonatal, paediatric and adult specialist retrieval teams utilising road, rotary and fixed wing platforms. MedSTAR is accessed through a single number utilising a central clinical coordination centre within South Australian Ambulance Service with nurse retrieval coordinators and a medical retrieval consultant 24 hours a day. MedSTAR supports a population of 1.7 million over 1 million square kilometres performing ~2800 missions annually. Retrieval teams are located at one central base collocated with the air assets at Adelaide airport to ensure timely and efficient response. MedSTAR provides support to rural clinicians and facilitates telemedicine consultations to improve clinical decision making to ensure we get the right patient to the right place in the right time with the right team.

Oslo HEMS
The Air Ambulance Department, Oslo University Hospital, covers about 2 mill of population of Norway (2.8 mill for ICU-transfers) and has part of a national responsibility for retrievals with iNO and ECMO. On helicopters, fixed wing and road vehicles neonates, children and adults are transported in a consultant delivered service with more than 1000 transports annually of ventilated patients. Norwegian Medical Response Team for Highly Infectious Diseases which is a national service offering transfer by special designed ICU-ambulance and by ground or air (C-130 Hercules) is also part of the department.

DRF Stiftung Luftrettung gAG
DRF Luftrettung is a German non-profit HEMS provider operating 31 bases in Germany, founded 1972. Currently we use rotary as well as fixed wing platforms for primary missions as well as for retrievals.  In 2019 we did more than 40.000 missions with a prehospital team comprising a physician and a paramedic including winch operation, ECMO- and neonatal incubator transfers and night missions.

RACQ Lifeflight Rescue / LifeFlight Retrieval Medicine
RACQ Lifeflight Rescue provides critical care aeromedical retrievals for the state of Queensland and internationally. Founded in 1979, the not for profit organisation conducts more than 6000 missions per year in conjunction with Retrieval Services Queensland. The service operates from nine bases with ten rotary wing assets and three air ambulance jets. The critical care teams comprising paramedics, nurses and physicians deliver prehospital trauma care including winch operations as well as inter facility and international retrievals.

## Centre for pre-hospital emergency care, Oulu University Hospital

The Finnish HEMS system is managed by a national administrative company FinnHEMS Ltd. and there are six HEMS units available 24/7. Five HEMS units are physician-staffed and the northernmost unit is manned with advanced-level paramedics. Three southern bases use Airbus H135 and three northern bases use Airbus H145 helicopters. A rapid response car (RRC) is available in every base for short-range missions and for poor weather conditions. The HEMS units in Finland are dispatched via Finnish Emergency Response Centre (112 Finland). HEMS units are dispatched to all high-risk medical situations based on the provision of medical equality in Finland.

ScotSTAR Emergency Medical Retrieval Service (EMRS)
EMRS provides secondary critical care retrieval to adults in remote and rural Scotland, as well as primary prehospital critical care to the c entire population. There are two bases: one in Glasgow, and one in Aberdeen, utilising fixed- and rotary-wing aircraft, and road vehicles. All missions are staffed by a consultant in emergency medicine, intensive care, or anaesthetics, and either a Retrieval Practitioner (from a nurse or paramedic background) or a junior doctor in training with the Service.
