# Supplementary material for: Decision support tool and suggestions for the development of guidelines for the helicopter transport of patients with COVID-19
Source: Scand J Trauma Resusc Emerg Med. 2020 May 25;28:43. doi: 10.1186/s13049-020-00736-7 (PMC7247287; doi:10.1186/s13049-020-00736-7)
Supplement: Supplementary file 2 — Additional file 2. [file 13049_2020_736_MOESM2_ESM.docx]

# Appendix B

A literature search was performed on February 20th in PubMed/MEDLINE (from 1966 through January 2020) with the following terms:

- (“Aeromedical evacuation” OR “transportation of patients” OR “air ambulance” OR “HEMS” OR “Helicopter”) AND (“ebola” OR “lassa” OR “viral hemorrhagic” OR “highly infectious” OR “highly hazardous” OR “contagious” OR “communicable” OR “Middle East respiratory syndrome (MERS)” OR “SARS” OR “smallpox”)*
- “Air Ambulances"[Mesh]) AND "Infection Control"[Mesh]
- "Aeromedical” AND "Infection Control"[Mesh]
- "SARS Virus"[Mesh] OR "Middle East Respiratory Syndrome Coronavirus"[Mesh]) AND ("Air Ambulances"[Mesh] OR aeromedical[All Fields])

A similar search strategy was used by authors of a previous literature review of aeromedical transport of patients with highly contagious infectious disease.

Authors screened abstracts for the following inclusion criteria: peer-reviewed literature, written in English, which describes the aeromedical transport of persons with a highly hazardous communicable disease. Articles were excluded if they dealt with retrieval of patients by a transfer team specialised in containment of highly contagious infectious disease, or with the use of isolation tents or other specialist equipment.

The definition of a highly hazardous communicable disease is understood to include easily transmissible emerging infectious diseases.

124 papers were identified

30 were identified as relevant by the above criteria (24 after exclusion of duplicates)

After review of abstracts: exclusions -

·       5 excluded: no abstract

·       5 excluded: not aeromedical

·       8 excluded: use of an isolation tent or pod

·       3 excluded: not specific to highly contagious disease

·       2 excluded: not in English

·       1 excluded: purely editorial

A 2019 literature review (b) identified 14 studies pertinent to the aeromedical transport of patients with highly contagious infectious disease, by specialised teams using a high-level containment transport system. This review noted that none of the papers described in detail the PPE ensemble, nor the donning and doffing protocol. Details on waste disposal and aircraft decontamination were also limited.

In order to capture the emerging literature on transfer of COVID-19 patients, and how this might differ from other highly-contagious diseases, on 20 March 2020 we searched the same databases for

“COVID-19” AND "Airway Management"[MeSH] OR "Critical Care"[MeSH]  OR "Patient Transfer"[MeSH].

This search strategy was designed to include articles not specific to the aeromedical environment.

18 articles were identified. Four were identified as being relevant following review of abstracts.

- One article on the transfer of COVID-19 patients does not encompass aeromedical transfers, but concurs with our recommendations on ground transport[1] (REF transport Liew)
- Two editorial articles advise on how hospitals can prepare to transfer COVID patients; their recommendations have been included where relevant[2, 3]
- An editorial outline the preparations needed in a hospital system, but their relevance to retrieval is limited[4]

References

- 1. Liew MF, Siow WT, Yau YW, See KC: Safe patient transport for COVID-19. Critical Care 2020, 24(1):94.
- 2. Bouadma L, Lescure FX, Lucet JC, Yazdanpanah Y, Timsit JF: Severe SARS-CoV-2 infections: practical considerations and management strategy for intensivists. Intensive Care Med 2020, 46(4):579-582.
- 3. Qiu H, Tong Z, Ma P, Hu M, Peng Z, Wu W, Du B: Intensive care during the coronavirus epidemic. Intensive Care Med 2020, 46(4):576-578.
- 4. Liew MF, Siow WT, MacLaren G, See KC: Preparing for COVID-19: early experience from an intensive care unit in Singapore. Crit Care 2020, 24(1):83.
