# Supplementary material for: Decision support tool and suggestions for the development of guidelines for the helicopter transport of patients with COVID-19
Source: Scand J Trauma Resusc Emerg Med. 2020 May 25;28:43. doi: 10.1186/s13049-020-00736-7 (PMC7247287; doi:10.1186/s13049-020-00736-7)
Supplement: Supplementary file 3 — Additional file 3. [file 13049_2020_736_MOESM3_ESM.docx]

# Appendix C

## Intubation

Have a low threshold for intubating the patient[1]. Intubated patients may have a lower risk of aerosol contamination of the aircraft than other respiratory support systems (NIV,facemask). If the patient coughs a lot when on a non-rebreather mask, consider intubation. The decision to prophylactically intubate the patient in such cases must weigh up their predicted clinical course against the inevitable pressure on ventilated ICU beds. Intubation is potentially an aerosolizing procedure, and so should be undertaken with proper precautions. Planning should focus on minimizing the number of staff, and the amount of equipment which contacts the infected patient[1, 2].Remember that your personal protection is the priority[3].Utilise full PPE, including double gloving, when managing the airway of any suspected or confirmed case of COVID-19[2, 4].

## Personnel

- Intubation by a sole operator may be appropriate. Use the most experienced intubator[1, 2, 5].
- Utilise competent clinical staff who have already been in contact with the patient as an airway assistant
- Position the airway assistant behind the intubator to minimize their exposure
- One clean person as a spotter and “runner” for additional equipment

## Equipment

- Consider placing a high-efficiency particulate air (HEPA) filter on the expiratory limb of the ventilator circuit to protect the ventilator. Ventilator manufacturers are providing advice on where to position filters, depending on the position of the expiratory valve
- Bag/valve/mask (BVM) with an HEPA filter and a positive end-expiratory pressure (PEEP) valve
- Attach a closed in-line suction set-up to the ventilator circuit prior to induction. Portable suction units may require a high-performance viral filter to be fitted
- Perform a kit dump in a clean area, on a procedure trolley that can be taken into the patient’s room
- Use disposable equipment where possible. Consider using stylet rather than bougie due to the potential elastic recoil of the bougie flinging droplets
- Prepare standard medications for induction, ongoing sedation and rescue blood pressure management prior to entering the room
- Have a specific clear waste bag for any non-disposable equipment which will need to be decontaminated

## Intubation procedure

- Discuss the airway plan prior to applying PPE, and plan for increased difficulty with communication while wearing PPE
- The use of video laryngoscopy is advocated in order to keep the face of the intubator furthest away from the airway[2]
- NIV and high flow nasal oxygen for pre-oxygenation should be avoided as it may aerosolize the virus[6]
- Avoid awake fiberoptic intubation unless specifically indicated, atomized local anaesthetic may aerosolize the virus[7]
- Plan the timing of moving the patient to the transport stretcher. It will be easier to package them if they are moved prior to intubation, but movement of the conscious patient may trigger coughing and require multiple assistants
- Turn off oxygen at the wall before removing the patient’s oxygen mask to avoid aerosolizing droplets
- Consider suxamethonium as paralytic to minimize apnea time as BVM should be avoided, if the operator is familiar with this drug. Remove outer set of gloves and use them to cover disposable laryngoscope prior to putting it in the bin immediately after placing the endotracheal tube(ETT)
- Be prepared for rapid desaturation of the patient. If manual ventilation prior to intubation is absolutely necessary use small tidal volumes and a two-handed seal if needed[1, 2]

## Post intubation

- Transfer the patient immediately to the ventilator, or clamp the tube prior to disconnecting the BVM to avoid passive exhalation
- Discard all opened kit
- N95/P2 masks should be discarded immediately after leaving the room where the intubation took place[8]
- If called to transfer a patient already on a ventilator, the ventilator should be paused and the ETT clamped prior to connection to the transport ventilator
- Ensure that suction units have HEPA filters on the air-outlet side if the manufacturer cannot guarantee that this air is pathogen-free

## References

1. Peng PWH, Ho P-L, Hota SS: Outbreak of a new coronavirus: what anaesthetists should know. Br J Anaesth 2020.

2. Wax RS, Christian MD: Practical recommendations for critical care and anesthesiology teams caring for novel coronavirus (2019-nCoV) patients. Can J Anaesth 2020.

3. Cheung JC, Ho LT, Cheng JV, Cham EYK, Lam KN: Staff safety during emergency airway management for COVID-19 in Hong Kong. The Lancet Respiratory medicine 2020.

4. Casanova LM, Rutala WA, Weber DJ, Sobsey MD: Effect of single- versus double-gloving on virus transfer to health care workers' skin and clothing during removal of personal protective equipment. Am J Infect Control 2012, 40(4):369-374.

5. Airway Management in patients suffering from COVID-19 [http://www.siaarti.it/SiteAssets/News/COVID19%20-%20documenti%20SIAARTI/SIAARTI%20-%20Covid-19%20-%20Airway%20Management%20rev.1.1.pdf]

6. Caputo KM, Byrick R, Chapman MG, Orser BJ, Orser BA: Intubation of SARS patients: infection and perspectives of healthcare workers. Can J Anaesth 2006, 53(2):122-129.

7. Huang C, Wang Y, Li X, Ren L, Zhao J, Hu Y, Zhang L, Fan G, Xu J, Gu X et al: Clinical features of patients infected with 2019 novel coronavirus in Wuhan, China. Lancet 2020, 395(10223):497-506.

8. P2/N95 mask fit checking [https://www.health.qld.gov.au/clinical-practice/guidelines-procedures/diseases-infection/infection-prevention/transmission-precautions/p2n95-mask]
